# Spectrally separated dual-label upconversion luminescence lateral flow assay for cancer-specific STn-glycosylation in CA125 and CA15-3

**DOI:** 10.1007/s00216-024-05275-z

**Published:** 2024-04-08

**Authors:** Miikka Ekman, Teppo Salminen, Kirsti Raiko, Tero Soukka, Kamlesh Gidwani, Iida Martiskainen

**Affiliations:** Biotechnology Unit, Department of Life Technologies, Faculty of Technology, University of Turku, Turku, Finland

**Keywords:** Spectral multiplexing, Upconversion luminescence, Lateral flow assay, Glycovariant assay, Ovarian cancer

## Abstract

Multiplexed lateral flow assays (LFAs) offer efficient on-site testing by simultaneously detecting multiple biomarkers from a single sample, reducing costs. In cancer diagnostics, where biomarkers can lack specificity, multiparameter detection provides more information at the point-of-care. Our research focuses on epithelial ovarian cancer (EOC), where STn-glycosylated forms of CA125 and CA15-3 antigens can better discriminate cancer from benign conditions. We have developed a dual-label LFA that detects both CA125-STn and CA15-3-STn within a single anti-STn antibody test line. This utilizes spectral separation of green (540 nm) and blue (450 nm) emitting erbium (NaYF_4_:Yb^3+^, Er^3+^)- and thulium (NaYF_4_: Yb^3+^, Tm^3+^)-doped upconverting nanoparticle (UCNP) reporters conjugated with antibodies against the protein epitopes in CA125 or CA15-3. This technology allows the simultaneous detection of different antigen variants from a single test line. The developed proof-of-concept dual-label LFA was able to distinguish between the ascites fluid samples from diagnosed ovarian cancer patients (*n* = 10) and liver cirrhosis ascites fluid samples (*n* = 3) used as a negative control. The analytical sensitivity of CA125-STn for the dual-label LFA was 1.8 U/ml in buffer and 3.6 U/ml in ascites fluid matrix. Here we demonstrate a novel approach of spectrally separated measurement of STn-glycosylated forms of two different cancer-associated protein biomarkers by using UCNP reporter technology.

**Graphical Abstract:**

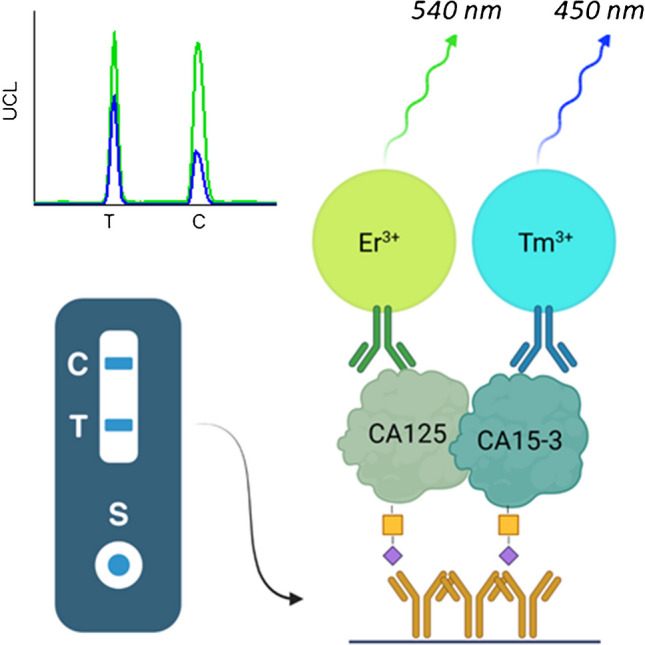

## Introduction

Lateral flow assays (LFAs) are simple, easy-to-use diagnostic tests that provide information quickly. Because of these features, they are often used in point-of-care and resource-limited settings instead of sample analysis in a central laboratory. Conventional LFAs rely on visual detection and are typically used in infectious disease and pregnancy testing. However, recently there has been a growing interest in shifting from conventional LFAs towards luminescent LFA technology, and thus expanding the scope of traditional LFAs. By using luminescent nanoparticle reporters instead of visually detectable nanoparticles, the LFA detection sensitivity can be increased in infectious disease testing [1–3]. Quantitative detection of certain analytes in LFA format can provide a simple and affordable solution to be used in point-of-care settings [4, 5]. Luminescent LFA reporter technology combined with a portable reader instrument serves as a promising alternative for a point-of-care testing platform. Reader-based point-of-care testing systems are becoming more and more popular and accepted for disease testing in resource-limited settings (REASSURED criteria) [6]. The main focus of LFA testing has been in infectious disease testing but there is a growing interest of cancer testing in this format [7] as the global disease profile is changing [8].

Cancer diagnostics in clinical practice is a complicated procedure and the diagnosis made by the physician is often based on many methods such as evaluation of physical symptoms, imaging, biopsies, and blood testing. Blood testing is a desired method because of its less invasive nature and easiness in clinical practice. However, many blood-based cancer biomarkers suffer from poor specificity for a certain cancer type and often it is difficult to distinguish between malignant and benign conditions based on blood protein biomarkers. Alteration in protein glycosylation during posttranslational modification is a common feature in tumor cells [9, 10]. Some cancer biomarkers have cancer-specific glycosylations which improve the specificity of the glycovariant assay in contrast to traditional biomarker assay [11]. Furthermore, multiparameter assays for more than one biomarker can provide the physician with more information regarding the status of the patient. This has been shown potentially to improve the diagnostic accuracy, e.g., in ovarian cancer [12, 13].

Epithelial ovarian cancer (EOC) is the most lethal form of gynecological malignancies [14]. EOC symptoms are often vague and unspecific, appear often in later stages, and are associated with many other non-malignant conditions. Because of this, EOC is commonly diagnosed at late stage; the relative survival rate at 5 years is globally around 30–40% [15]. Early diagnosis of EOC could increase the 5-year survival rate up to 90% [16]. Currently, EOC diagnosis is based on imaging techniques and laboratory tests but these methods lack in sensitivity and accuracy. Present laboratory diagnostics of EOC mostly rely on serum biomarker CA125 which is used as a tool for differential diagnosis and for monitoring disease progression and therapy response [17]. However, serum CA125 concentration is commonly elevated in several benign gynecologic conditions such as endometriosis [18]. CA125 is also present in elevated concentrations in many other malignant carcinomas [19, 20]. Alterations in CA125 glycosylation, particularly the expression of the sialyl-Tn antigen (STn), are shown to be a potential biomarker for the early and specific detection of EOC, and the detection of sialylated CA125 improves the diagnostic performance of the immunoassay in contrast to the traditional immunoassay against CA125 protein epitopes [21]. In addition, another cancer-associated antigen CA15-3 with STn glycosylation has been shown to differentiate early-stage EOC from healthy and benign subjects [22]. Measurement of glycosylated forms of CA15-3 and CA125 in parallel could potentially improve the accuracy of EOC diagnostics.

Simultaneous on-site measurement of different substances from a single sample is a desired feature in many fields of application, e.g., drug testing and clinical diagnostics, for achieving efficient and high-throughput analysis. Multiplexed LFA can be used for the detection of multiple biomarkers within one test strip and it has several benefits, including improving the efficiency of testing and reducing costs. Multiparameter rapid testing is particularly desired for those on-site applications in which advanced decision-making is requested or availability of samples is limited [23]. LFA test strip architecture inherently enables aligning more than one detection site in a single analytical device. The spatial separation of multiple detection sites is easily obtained by applying several test lines or dots within the nitrocellulose membrane and is up to date the most popular way to conduct multiplexing in LFA [23, 24]. However, result interpretation of a multi-line LFA is not as simple as that of a conventional single-line LFA, particularly when the read-out is visual. Another approach for multiplexing LFAs is using individual strips arranged in an array-like format, which can be carried out in different cartridge designs built around the conventional single-line test strip [25]. Sometimes, spectral separation by reading the signal of two different reporters within a single test line is an elegant and feasible way of multiparameter detection in LFA. Spectrally multiplexed LFAs have been developed with different reporter technologies including enzyme labels [26], quantum dots (QDs), and sometimes with mixed-color visual detection [27, 28]. Spectral multiplexing approach has been previously successfully demonstrated in simultaneous detection of two different tumor markers, alpha fetoprotein (AFP) and carcinoembryonic antigen (CEA), by using multicolor QDs [29].

**Fig. 1:**
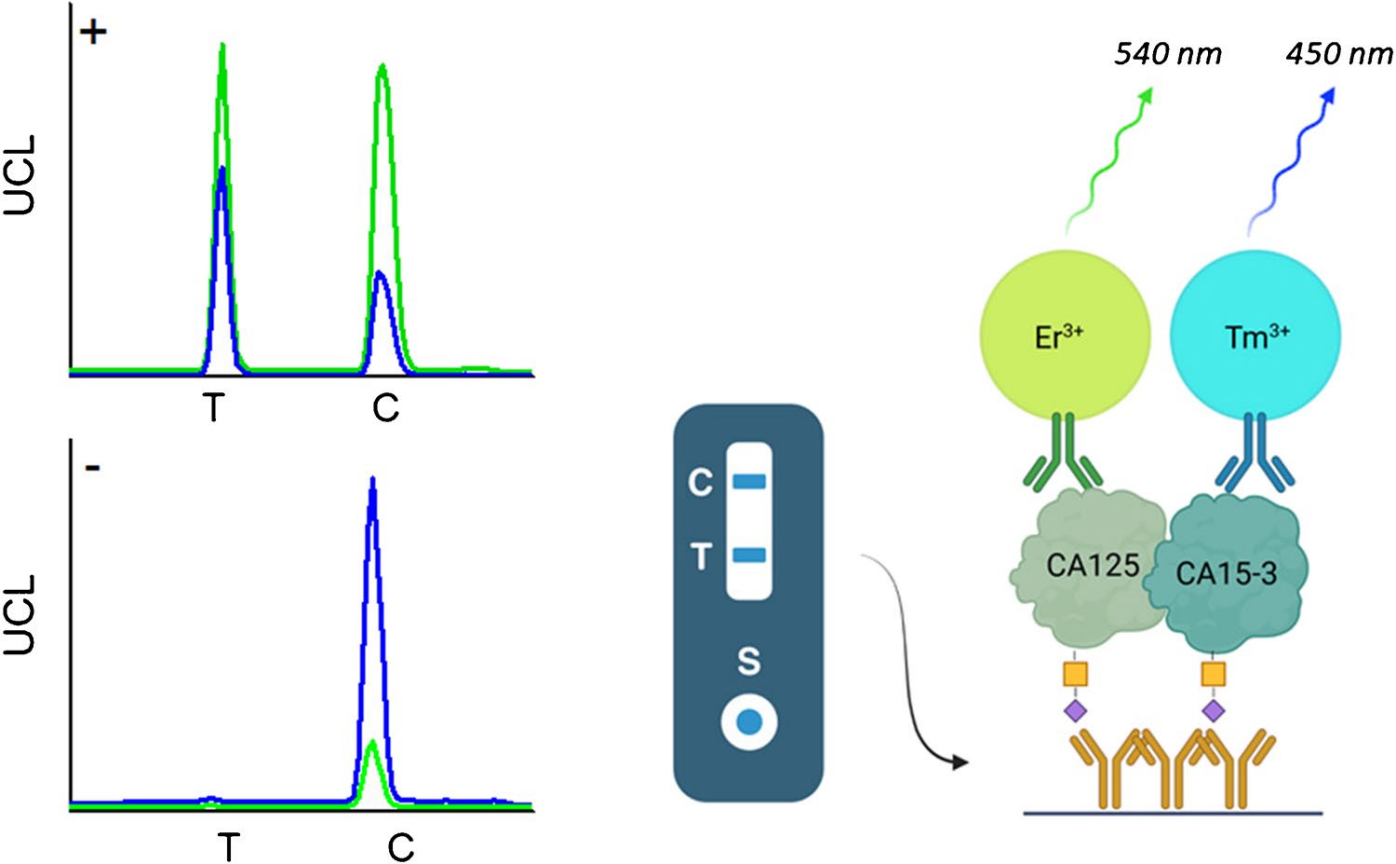
Working principle of the spectrally separated dual-label LFA. Antibodies recognizing cancer-specific STn-glycosylation are immobilized on the test line (T). STn-glycovariants of cancer-associated biomarkers CA125 and CA15-3 are detected with erbium (Er^3+^) and thulium (Tm^3+^) doped UCNPs conjugated with antibodies against the protein epitopes. Illustration created with BioRender

We have established a dual-label LFA for the simultaneous detection of STn-glycosylated ovarian cancer–associated antigens CA125 and CA15-3 by using spectrally separated dual-color upconversion luminescence (UCL) measurement (Fig. 1). Upconverting nanoparticles (UCNPs) were selected as reporters as they have narrow emission peaks in optically separable wavelengths, Tm-doped UCNPs at 450 nm and Er-doped UCNPs at 540 nm. UCNPs are also stable and do not photobleach and due to the anti-Stokes phenomenon, which eliminates the background autofluorescence originating from the components of the test device or sample matrix, they are applicable for high-sensitivity applications [30]. UCNPs have previously been used successfully in many LFAs with highly sensitive detection, including the detection of STn-glycosylated CA125 [31].

In this study, the UCNP reporters were conjugated with antibodies targeted against CA125 and CA15-3 protein epitopes. STn-glycosylated forms of these proteins were captured by anti-STn antibody immobilized within the test line on LFA nitrocellulose membrane. Here we demonstrate the use of two different color UCNPs in dual-parameter measurement within a single test line in an LFA test device.

## Materials and methods

### Materials and reagents

Monoclonal anti-CA125 antibody (4602) was provided by Medix Biochemica Oy (Espoo, Finland). Monoclonal anti-CA15-3 antibody (Ma552), monoclonal anti-STn antibody (STn1242), and the CA125-STn glycovariant isolated from NIHOVCAR-3 cell line (OVCAR3-CA125) used as a calibrator material were provided by Fujirebio Diagnostics AB (Sweden). Rabbit anti-mouse IgG (RAM) was purchased from Invitrogen (MA, USA).

Backing card (Standard Grade Backing Laminate) and cover tape (KN-CPP1-Clear Kenosha cover plastic) were purchased from Kenosha Tapes (Amstelveen, Netherlands). Nitrocellulose (LFNC-C-BS023-70) was purchased from Nupore Filtration Systems Pvt. Ltd. (Ghaziabad, India). Glass fiber sample pad 8951 was purchased form Ahlstrom-Munksjö Oyj (Helsinki, Finland) and cellulose absorbent pad CFSP223000 was purchased from Merck Millipore (Burlington, MA, USA).

### UCNP synthesis and characterization

Er-UCNPs (NaYF_4_: 17% Yb^3+^, 3% Er^3+^) and Tm-UCNPs (NaYF_4_: 20% Yb^3+^, 0.5% Tm^3+^) were synthesized in-house with high-temperature co-precipitation in organic oils as described earlier [32]. The oleic acid–capped UCNPs were characterized by transmission electron microscopy (TEM), by dispersing to 0.3 mg/ml in toluene and applying 3 µl on 300-mesh carbon-formvar-coated copper grid (Agar Scientific, Essex, UK), and imaging with JEM-1400 Plus (JEOL, MA, USA) under 80-kV electron beam. UCNP size was measured from TEM images with semi-automatic in-house program based on setting color shade thresholds. Emission spectra of UCNPs were measured with a Varian Cary® spectrophotometer modified with a 980-nm excitation laser source from 1 mg/ml dilutions of oleic acid–capped UCNPs in DMSO. The UCNPs were polyacrylic acid (PAA, Mw 2000) coated and bioconjugated following a previously published protocol with a small modification [33]. Er-UCNPs were conjugated with anti-CA125 Mab 4602 and Tm-UCNPs were conjugated with anti CA15-3 Mab Ma552 using EDC/sulfo-NHS chemistry. For anti-CA125, the EDC/sulfo-NHS activation was done with 2.5 mM EDC and for anti-CA15-3 the activation was done with 12.5 mM EDC.

### Samples

Ascites fluid from 10 ovarian cancer patients and 3 liver cirrhosis patients were obtained from Turku University Hospital. The Ethics Committee of the Hospital District of Southwest Finland and the University of Turku, Turku, Finland, approved the use of clinical materials applied (ClinicalTrials.gov identifier NCT01276574). The samples were previously measured with CanAg CA125 EIA and CanAg CA15-3 EIA from Fujirebio and in-house glycovariant microwell assays as described earlier [21].

### Preparation of LFA strips

Nitrocellulose, width of 25 mm, was laminated on a backing card. Test line 500 ng/cm of anti-STn Mab STn1242 and control line 500 ng/cm RAM in printing buffer (10 mM Tris pH 8, 5% EtOH, 1% sucrose, 40 µg/ml cherry red) were dispensed onto nitrocellulose 10 mm from the front end of the membrane and 5 mm apart from each other, using a Linomat 5 printer (Camag, Muttenz, Switzerland). After printing, the cards were allowed to dry overnight at +35 °C. Sample pads (16 mm wide) were blocked by saturating with blocking buffer (10 mM borate buffer pH 7.5, 0.05% Tween-20, 1% BSA) and dried for a minimum of 2 h at +35 °C. The sample pad and 34-mm-wide absorbent pad were assembled on the membrane card and the nitrocellulose membrane was protected by clear cover tape. Cards were cut into 4.8-mm-wide strips and 100 ng/2 µl/strip UCNP conjugates in drying buffer (0.05 M Tris-HCl, pH 7.5, 0.04% NaN3, 2 mM KF, 1% BSA, 0.05% Tween-20, 0.5 M NaCl, 5% sucrose, 5% trehalose, and 0.05% PAA) were dried to the middle of the sample pad in a desiccator, at +35 °C for 1.5 h. Strips were stored at room temperature, protected from light and humidity.

### Dual-label lateral flow immunoassays

The general protocol for the LFAs was the following: First, 35 µl sample and 25 µl assay buffer (0.05 M Tris-HCl, pH 7.5, 0.04% NaN3, 2 mM KF, 1% BSA, 0.05% Tween-20, 0.5 M NaCl, and 0.05% PAA) were added to a microtiter well, followed by the placement of the LF strip containing dried UCNPs to the well. The strip was allowed to absorb the liquid for 9 min and then moved to a separate microtiter well containing 50 µl of washing buffer (10 mM Bis-Tris, pH 6.5, 250 mM NaCl, 1% BSA, 0.5% Tween-20, and 2 mM KF). The wash buffer was allowed to run 21 min and then the strips were measured after total assay time of 30 min with an UCL reader device (Upcon, Uniogen Oy, Finland). The UCL signals from test line and control line for CA125-STn detection (Er-UCNPs) were measured at 540 nm and for CA15-3 detection (Tm-UCNPs) at 450 nm. The nitrocellulose membrane was scanned (160 scan points over the range of 25 mm, 1 mm emission spot size, and 100 ms measurement time) with 976-nm excitation laser using 100% relative laser power. The average baseline signal before and after test line was subtracted from the maximum signal of the test line [2].

For determining the suitability of measuring both CA125-STn and CA15-3-STn from a single anti-STn test line, three liver cirrhosis ascites fluid samples (LC) and ten ascites fluid samples from ovarian cancer patients (OvCa) (diluted 1:10 in 7.5% BSA-TSA) were measured with dual-label LFA containing dried reporters for the detection of both CA125-STn and CA15-3-STn, ‘singleplex’ CA125-STn LFA and ‘singleplex’ CA15-3-STn LFA. The discrimination between the LC and OvCa samples was calculated based on the cutoff signal level that was obtained from the average signal plus 3xSD from the LC samples.

### Analytical sensitivity of the dual-label LFA for CA125-STn

The immunoassay analytical sensitivity of the dual-label LFA was determined as defined in the literature [34] with CA125-STn calibrator spiked in pooled LC sample or in 7.5% BSA-TSA buffer within the concentration range of 1–2500 U/ml. The 0 U/ml calibrator samples were assayed in 12 replicates and the concentrations 1–2500 U/ml were assayed in 6 replicates each. The cutoff for analytical sensitivity was calculated as the average of the zero concentration plus 3xSD. The experiment was done only with CA125-STn, as there was no CA15-3-STn calibrator available.

### Interference study

Potential interference from high CA125 concentration at the anti-STn test line with the CA15-3 measurement was studied. Ascites samples of ovarian cancer patients were spiked with 1000 U/ml of CA125-STn calibrator. The samples were assayed with the dual-label LFA and measured for both CA125-STn and CA15-3-STn.

### Crosstalk between Er^3+^and Tm^3+^ UCNPs

Crosstalk between Er^3+^ and Tm^3+^ UCNPs was determined by measuring UCL from test line containing only either Er^3+^-UCNPs or Tm^3+^-UCNPs for both Er and Tm channel. The Er channel was filtered with a bandpass filter (545 nm, bandwidth 7 nm) and an IR-filter provided by Uniogen. The Tm channel was filtered with a bandpass filter (460 nm, bandwidth 20 nm) from Hidex Sense reader (Hidex Oy, Finland).

## Results

**Fig. 2:**
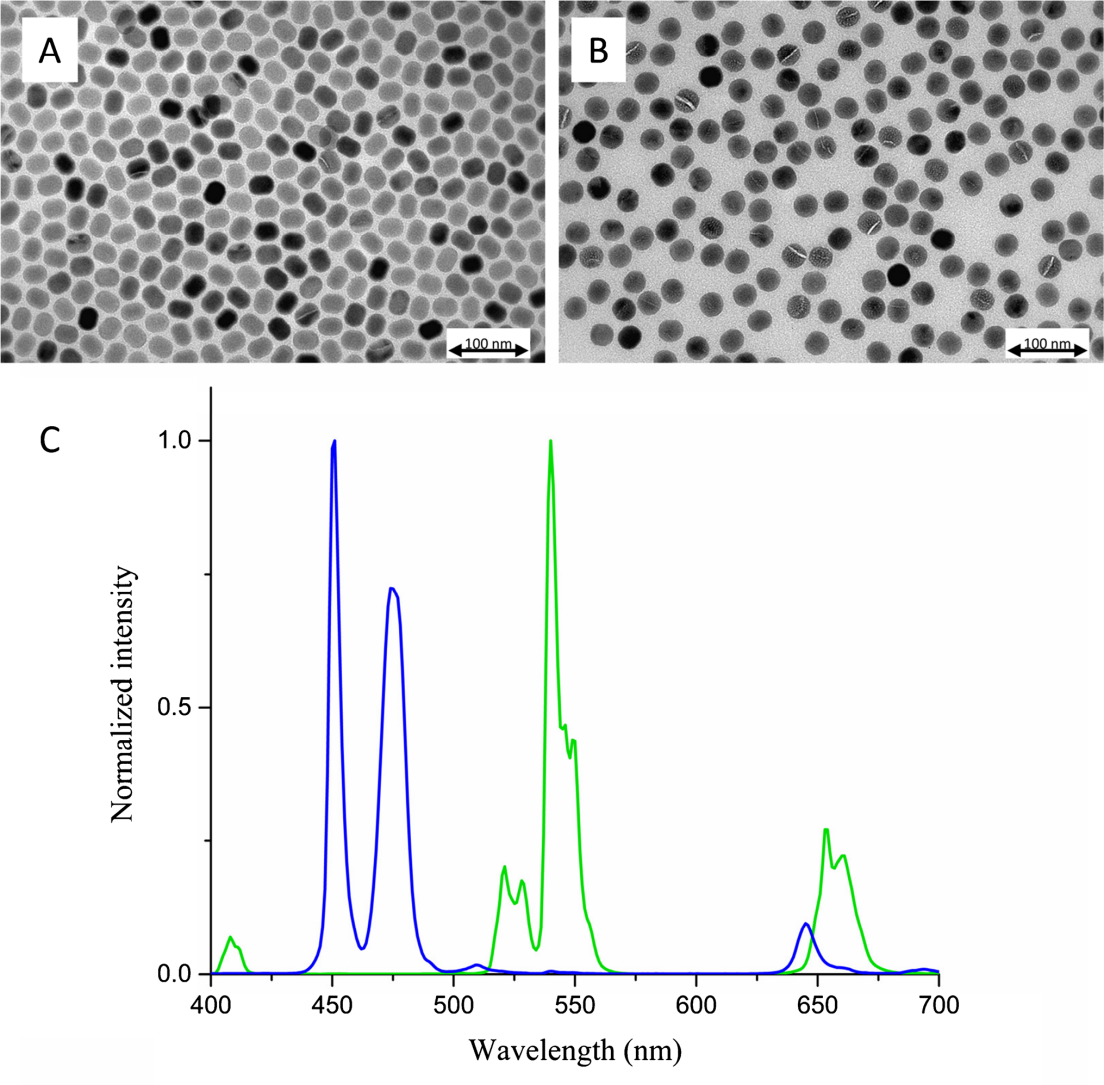
TEM images of non-functionalized Er^3+^-UCNPs in toluene (**A**) and Tm^3+^-UCNPs in toluene (**B**), under 80,000× magnification, and their respective emission spectra (**C**) in DMSO, under 980-nm excitation, both normalized by their highest intensity emission peaks, which for Er-UCNP was at 540 nm and for Tm-UCNP at 450 nm

The UCNPs were characterized and based on the TEM images (Fig. 2A and B); the average diameters of the Er- and Tm-doped UCNPs were 25.8 ± 1.7 nm and 27.8 ± 0.8 nm, respectively. In the emission spectra measurements (Fig. 2C), there was no significant overlapping of the measured spectra within the measurement range. In the strip reader, the measured crosstalk of Er-signal to Tm-channel was 0.30%, and that of Tm-signal to Er-channel was 0.16%.

**Fig. 3:**
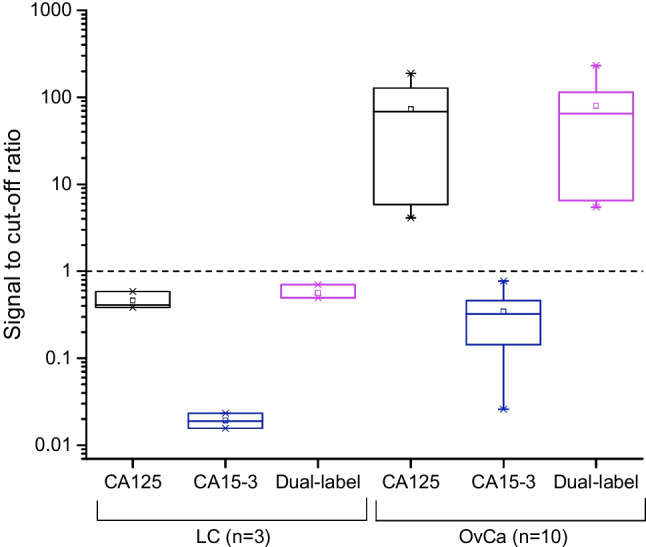
Measurement of CA125 (erbium emission at 540 nm) with CA125-STn LFA (black), CA15-3-STn LFA (blue), and dual-label LFA (purple). Liver cirrhosis (LC) ascites fluid samples (*n* = 3) were used as negative controls and they were assayed with ovarian cancer (OvCa)–positive ascites fluid samples (*n* = 10)

**Fig. 4:**
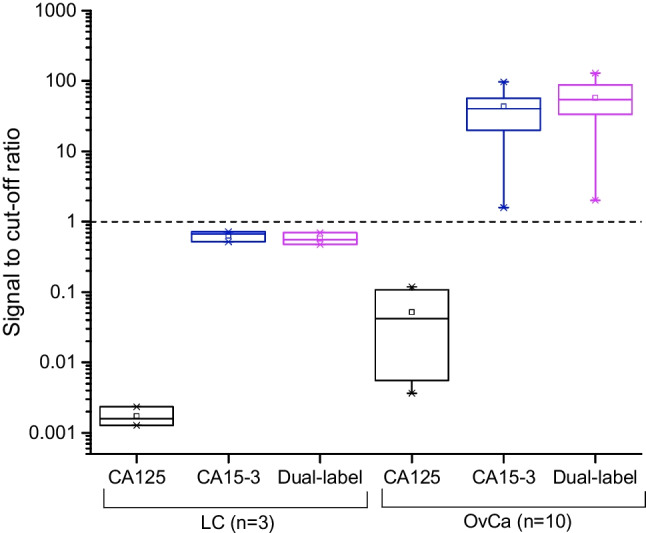
Measurement of CA15-3 (thulium emission at 450 nm) with CA125-STn LFA (black), CA15-3-STn LFA (blue), and dual-label LFA (purple). Liver cirrhosis (LC) ascites fluid samples (*n* = 3) were used as negative controls and they were assayed with ovarian cancer (OvCa)–positive ascites fluid samples (*n* = 10)

The developed dual-label LFA was evaluated by assaying ascetic fluid samples from patients with diagnosed ovarian cancer (OvCa, *n* = 10) and ascetic fluid samples from liver cirrhosis (LC, *n* = 3) patients as negative control. The dual-label LFA was compared with the ‘singleplex’ LFA strips containing components only for the detection of either CA125-STn or CA15-3-STn. All the performed LFAs (dual-label LFA, CA125-STn LFA, and CA15-3-STn LFA) were measured by using a UCL reader either with emission measurement at 540 nm (CA125 detection) or at 450 nm (CA15-3 detection). In the case of CA125 measurement (Fig. 3), the ‘singleplex’ CA125-STn LFA and dual-label LFA showed similar results with both ovarian cancer–positive and ovarian cancer–negative ascites fluid samples. The signal measured from CA15-3-STn strips was below the cutoff level. Respectively, the CA15-3 measurement discriminated well between the positive and negative samples in the case of both the ‘singleplex’ CA15-3-STn LFA and dual-label LFA (Fig. 4). CA125-STn LFA strips did not give any interfering signal above the cutoff in CA15-3 measurement.

**Fig. 5:**
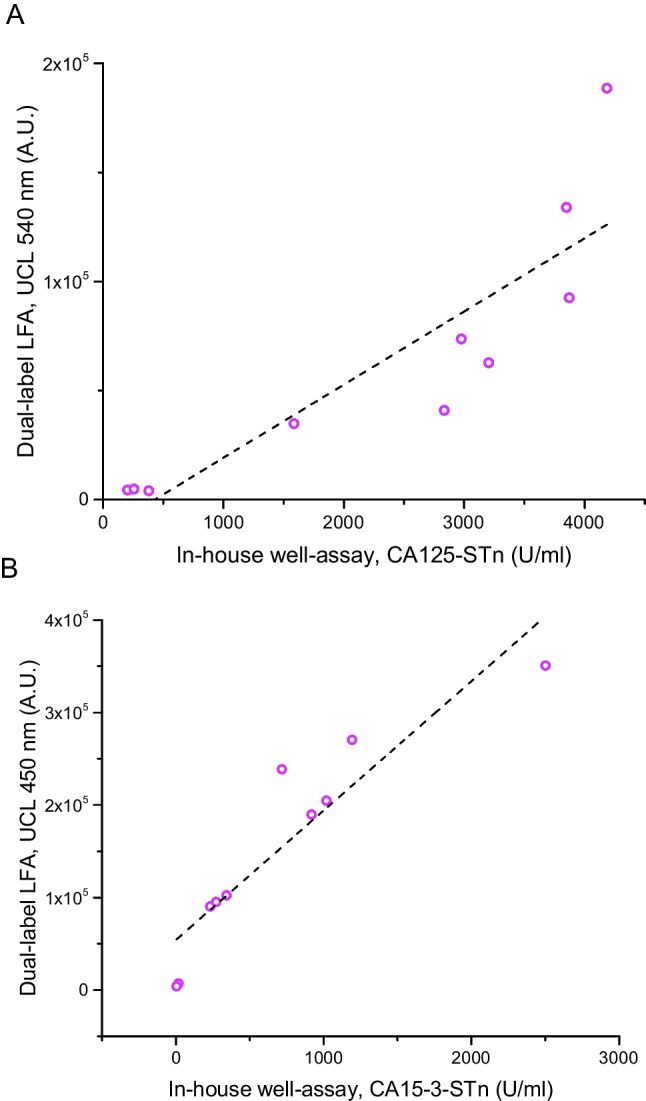
Method comparison between the dual-label LFA and in-house well-based assay for the detection of CA125-STn (**A**) (*R*^2^ = 0.77) and CA15-3-STn (**B**) (*R*^2^ = 0.85). The method comparison was done with the ovarian cancer ascetic fluid samples (*n* = 10)

A method comparison for the developed dual-label LFA and in-house well-based assay described earlier [21] is presented in Fig. 5. The correlation was good for both CA125 and CA15-3 measurements, with *R*^2^ values of 0.77 and 0.85, respectively.

**Fig. 6:**
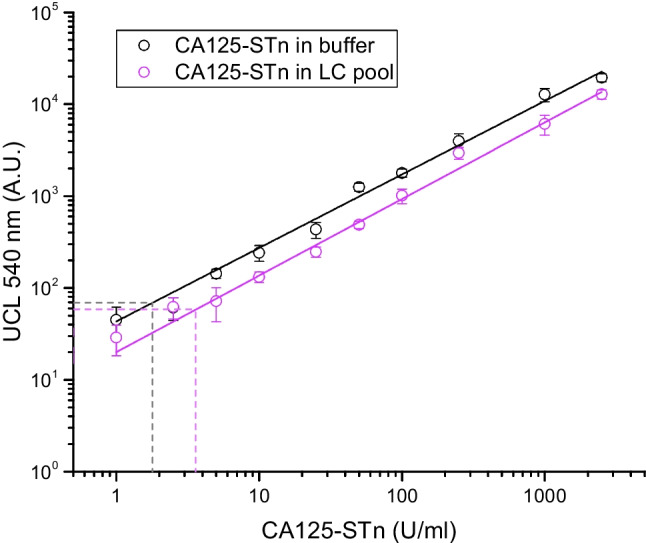
Calibration curve for the dual-label LFA with CA125-STn calibrator. The calibration curves were assayed with CA125-STn calibrator diluted either in buffer (black) or in liver cirrhosis (LC) ascites fluid pool (purple). The analytical sensitivities were 1.8 U/ml and 3.6 U/ml, respectively (dashed lines)

A standard series of CA125-STn calibrator material diluted in either buffer or LC pooled sample matrix was assayed with the dual-label LFA strips. The calibration curves for both matrixes are shown in Fig. 6. The analytical sensitivities, determined based on cutoff value calculated as the sum of average signal of the blank sample and 3xSD of the blank sample, were 1.8 U/ml for buffer-based system and 3.6 U/ml in LC ascites fluid.

**Fig. 7:**
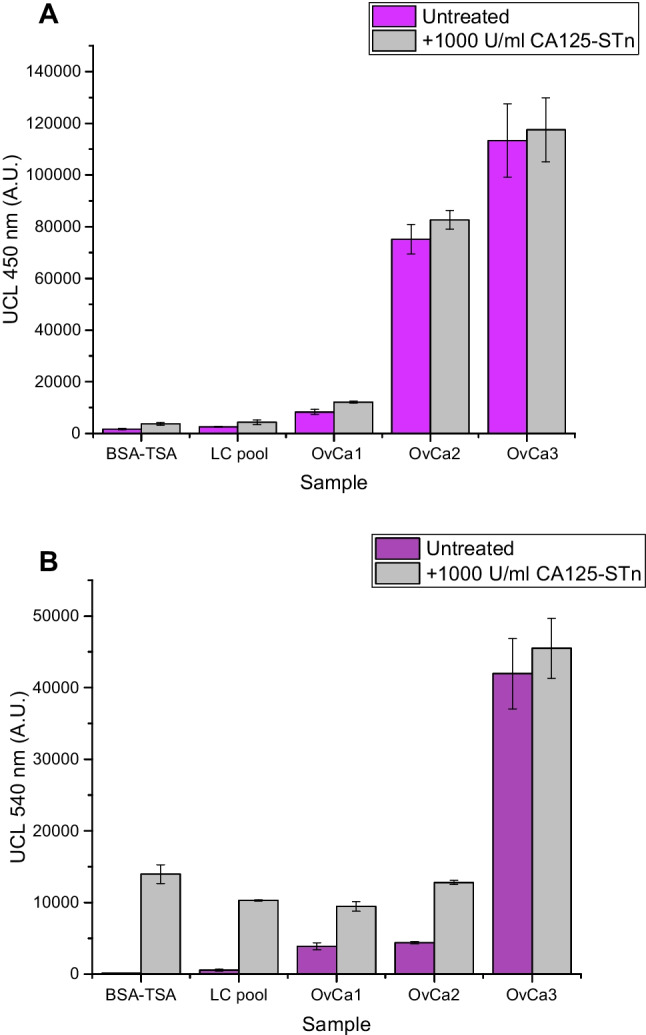
Comparison of untreated samples and samples spiked with 1000 U/ml CA125-STn antigen to study anti-STn test line saturation with CA125 when CA15-3 is measured. **A** Effect of spiked CA125-STn in CA15-3 measurement. **B** Effect of spiked CA125-STn in CA125 measurement

The possible interference of high concentrations of CA125 with the CA15-3 measurement was studied by spiking CA125-STn calibrator in the ovarian cancer–positive ascites fluid samples. The results shown in Fig. 7 indicate that this potential effect remains minimal as the signal levels did not significantly rise or decrease after the addition of CA125-STn antigen (Fig. 7A). As expected, the addition of CA125-STn to the samples increased the signal levels in the case of CA125 measurement (Fig. 7B).

## Discussion

The research shows a proof of concept of spectrally separated dual-label UCNP-LFA for the detection of aberrantly glycosylated cancer-associated biomarkers within one test line. The UCNPs were selected as LFA reporters as they are available in different colors within their emission spectra. Er-doped UCNPs emit light within green wavelength at around 540 nm and Tm-doped UCNPs have an emission peak at blue wavelength at 450 nm. Typically, the emission peaks of UCL are sharp and easily separable from each other and from the excitation wavelength, which is within the infrared area. This anti-Stokes phenomenon has been an advantage in creating highly sensitive immunoassays [33, 35]. The observed crosstalk of dual-label measurement was considered insignificant for the LFA results and was not further used as a correction factor for the measurements.

In the developed dual-label LFA, we used a single antibody within the test line. The used antibody was targeted against the STn-glycan structure, which has been observed to be present in both protein antigens CA125 and CA15-3, and this glycosylation pattern has been particularly associated with ovarian cancer. Glycovariant assays have been widely studied in cancer diagnostics, and they provide increased cancer specificity. The currently used detection methods relying only on detection components against protein epitopes of cancer-associated biomarker tend to be less specific, as the protein biomarkers are present in many benign conditions. Development of diagnostic assays for the glycovariant detection can provide more cancer-specific diagnostic tools for clinical practice.

As only one detection antibody was used at the test line, its capacity of binding excess amounts of STn-glycosylated proteins was studied. If the analyzed sample contains a vast amount of STn-glycosylated protein, it could interfere with the detection of other proteins. The effect of adding a clinically high concentration of CA125 in the ascites fluid samples was studied with the CA15-3 measurement. Addition of CA125 did not interfere with the CA15-3 measurement, as the signal levels obtained from the samples remained the same as in the untreated samples. When the presence of CA125 was measured, CA125 addition to the samples increased the signal levels as expected. The signal response after addition of 1000 U/ml was not completely linear. This may be because of a minor matrix effect occurring in the ascites samples, which is a typical phenomenon with real patient samples as the sample fluid contains an abundance of different biological compounds. The effect was not studied vice versa, as there was no CA15-3 calibration material available. We expect that the result would be similar also in this case, as the binding capacity of the test line was sufficient even with high concentrations of STn-glycosylated protein present.

In this study, we report the development of a quantitative and analytically sensitive assay to detect ovarian cancer–specific CA125-STn glycovariant. Sensitivity of the developed dual-label LFA was measured only with CA125 for the above-mentioned reason. The detection sensitivity was 1.8 U/ml in the buffer-based system and 3.6 U/ml in the ascites fluid sample matrix. The healthy range of circulatory CA125 concentration is commonly set below 35 U/ml. This threshold applies for the conventional CA125 protein assays but can be considered as directional for glycovariant assays as well. However, the concentration of STn-glycovariant is considered to be lower than the concentration of total CA125, and thus more sensitive assays are preferred for the glycovariant detection. Around 20% of ovarian cancer patients have CA125 levels between 35 and 100 U/ml, and 60% present with cancer at 100 U/ml or more [36]. The linear range of the developed dual-label LFA for the detection of CA125 was between 1 and 2500 U/ml. One out of five ovarian cancer patients does not produce CA125 (they have CA125 levels < 35 U/ml) [36], and these could be missed by screening only CA125 with the conventional detection methods. However, sensitive detection of glycovariant CA125 has been suggested to offer an opportunity for earlier detection of EOC particularly in postmenopausal women and early‐stage carcinomas [22].

The developed dual-label LFA was able to detect not only CA125-STn but also CA15-3-STn in the ascites fluid samples from diagnosed ovarian cancer patients. Here we demonstrate for the first time STn-glycovariant detection of CA15-3 in a 30-min rapid lateral flow test format. Both CA125 and CA15‐3 are reported to be elevated in patients with advanced EOC stage [37, 38]. It has been suggested that the sensitive detection of STn-glycosylated forms of CA125 and CA15-3 at very low biomarker concentrations could improve the early EOC detection [22, 39]. Based on these recent research findings, the simultaneous measurement of CA125 and CA15-3 STn-glycovariants could improve the diagnostic accuracy for early stage of the disease. The multiparameter analysis for other biomarker panels has been shown to improve the accuracy of ovarian cancer diagnosis [12, 13].

In this study, we used ascites fluid samples from ovarian cancer and liver cirrhosis patients to show the dual-label LFA’s ability to discriminate between the ovarian cancer cases and healthy control. For point-of-care use, the sample type should be easily collected like blood. To make the developed LFA suitable for point-of-care use, the study of blood samples should be applied. Previously, performance of the CA125-STn-LFA has been reported with serum samples [31].

The developed spectrally separated dual-label LFA serves as a proof of concept of using dual-color UCNPs detected at the same test line within one LFA strip. Similar multiplexing could be used for other applications requiring a biomarker panel assay for advanced decision-making. The approach could be expanded for example for infectious disease testing or for assaying a panel of cardiac markers [40–42]. The dual-color measurement could also enable ratiometric detection of two different variants of an antigen, and the scope of the technology could be expanded, e.g., for the detection of viral infections and virus variants. Multiplexing of an LFA by using a single test line enables keeping the LFA test device miniaturized as only a single standard-sized strip is required. Use of a single line also reduces the risk of invalid LFA runs, as the sample does not need to reach multiple lines at the farther distance within a nitrocellulose membrane.

Globally, 47% people have little or no access to sufficient diagnostic testing services. Due to remote locations, the accessibility of diagnostic testing at primary health care is a critical feature to guarantee equal access to diagnostics and correct treatment [43]. The developed dual-label LFA for STn-glycovariants of CA125 and CA15-3 is rapid to perform within 30 min, which suggests that after further validation with point-of-care compatible sample matrix, this kind of rapid test could be used for cancer screening at primary health care providing more people with access to diagnostic testing.

## Conclusion

As a conclusion, the developed dual-label LFA was able to distinguish STn-glycovariants of CA125 and CA15-3 within a single test line in ovarian cancer ascites fluid samples. The simultaneous detection of these biomarkers was achieved by using spectrally different color UCNP reporters. The measurement of Er and Tm emission signals from the single test line was feasible, and the crosstalk and interference in this system was minimal, thus suggesting that this developed dual-label LFA platform could be applied for different dual-biomarker assays.

## Declarations

### Source of biological material

Ascites fluid from 10 ovarian cancer patients and 3 liver cirrhosis patients were obtained from Turku University Hospital.
